# Clearinghouse Standards of Evidence on the Transparency, Openness, and Reproducibility of Intervention Evaluations

**DOI:** 10.1007/s11121-021-01284-x

**Published:** 2021-08-06

**Authors:** Evan Mayo-Wilson, Sean Grant, Lauren H. Supplee

**Affiliations:** 1 Department of Epidemiology and Biostatistics, Indiana University School of Public Health-Bloomington, 1025 E. 7th Street, Bloomington, IN 47405 USA; 2 Department of Social & Behavioral Sciences, Fairbanks School of Public Health, Indiana University Richard M, Indianapolis, IN USA; 3 Child Trends, Bethesda, MD USA

**Keywords:** Clearinghouse, Evidence standards, Open science, Reproducibility, Research transparency

## Abstract

Clearinghouses are influential repositories of information on the effectiveness of social interventions. To identify which interventions are “evidence-based,” clearinghouses review intervention evaluations using published standards of evidence that focus primarily on internal validity and causal inferences. Open science practices can improve trust in evidence from evaluations on the effectiveness of social interventions. Including open science practices in clearinghouse standards of evidence is one of many efforts that could increase confidence in designations of interventions as “evidence-based.” In this study, we examined the policies, procedures, and practices of 10 federal evidence clearinghouses that review preventive interventions—an important and influential subset of all evidence clearinghouses. We found that seven consider at least one open science practice when evaluating interventions: replication (6 of 10 clearinghouses), public availability of results (6), investigator conflicts of interest (3), design and analysis transparency (3), study registration (2), and protocol sharing (1). We did not identify any policies, procedures, or practices related to analysis plan registration, data sharing, code sharing, material sharing, and citation standards. We provide a framework with specific recommendations to help federal and other evidence clearinghouses implement the Transparency and Openness Promotion (TOP) Guidelines. Our proposed “TOP Guidelines for Clearinghouses” includes reporting whether evaluations used open science practices, incorporating open science practices in their standards for receiving “evidence-based” designations, and verifying that evaluations used open science practices. Doing so could increase the trustworthiness of evidence used for policy making and support improvements throughout the evidence ecosystem.

**Supplementary Information:**

The online version contains supplementary material available at 10.1007/s11121-021-01284-x.

Clearinghouses uniquely sit at the intersection of prevention science and evidence-based policy by supporting work to implement evidence-based preventive interventions at scale (Supplee & Meyer, 2015). Evidence-based policy involves the use of rigorous research to build credible evidence and focus resources on effective interventions (Baron, 2018). Clearinghouses are repositories of evidence that follow published standards to identify empirical studies of intervention effects, assess the validity and rigor of those studies, and disseminate information about “evidence-based” interventions (Burkhardt et al., 2015). Many clearinghouses rate research on preventive interventions for social, physical, and mental health, and academic problems (Neuhoff et al., 2015). They support evidence-based policy by “distilling findings from the most trustworthy research” (Mastri et al., 2015) to assist decision-makers in selecting interventions for which there is rigorous causal evidence (Office of Planning, Research, and Evaluation, 2018). In addition to providing incentives to use research evidence, clearinghouses also have ability to increase the use of evidence for decision making by building capacity and strengthening relationships with policymakers (Dumont, 2019).

Federally supported evidence clearinghouses are influential because they affect billions of dollars of funding for social programs (e.g., through legislation like the Families First Preventive Services Act)(Garcia et al., 2020; Maynard, 2018), and they influence standards for conducting and reporting intervention research (Buckley et al., 2021; Steeger et al., 2021). Previous studies have examined characteristics of evidence clearinghouses that are important to stakeholders in prevention science and allied disciplines (Burkhardt et al., 2015; Davies & Silloway, 2016). Other authors have raised concerns about the methodological hierarchies embedded in clearinghouse standards of evidence (Means et al., 2015; Valentine et al., 2017; Westbrook et al., 2017), the influence of socio-political context on clearinghouse designations of interventions as “evidence-based” (Fagan & Buchanan, 2016; Fagan et al., 2019; Gies et al., 2020), and variability in the utility of clearinghouse databases for stakeholders (Buckley et al., 2020; Gough & White, 2018; Harden et al., 2021; Neuhoff et al., 2015; Paulsell et al., 2017; Zack et al., 2019). This article is novel because it specifically examines the intersection between (1) federal clearinghouse standards of evidence and (2) transparent, open, and reproducible research practices. Although political and constituency considerations undoubtedly influence both federal clearinghouse standards and the use of evidence in policymaking, this article focuses on the potential impact of the methodological content of standards of evidence, including the connection between open science practices and public trust in intervention research that informs policy and practice (Lochman, 2021; Supplee & Meyer, 2015).

## Federal Clearinghouse Standards of Evidence Influence Research Design and Conduct

Clearinghouses evaluate research according to explicit standards of evidence. Using these standards, some clearinghouses rate interventions as “evidence-based” (or not), while others rate interventions according to several “tiers” of evidence (Means et al., 2015). For example, the Prevention Services Clearinghouse (PSC) evaluates interventions to support families and prevent foster care placements following standards that focus on the settings, methods, and results of eligible studies. PSC assigns one of four ratings: well-supported, supported, promising, or does not currently meet criteria (Wilson et al., 2019). These evidence tiers aim to distinguish the level of certainty that an intervention works and that evaluations produced true results.

In the United States (US), clearinghouses supported by the federal government are a subset of all clearinghouses that share several important characteristics. These clearinghouses are influential because investigators in prevention science and allied disciplines often design studies to meet federal clearinghouse standards of evidence. Federal recognition improves the visibility and perceived credibility of interventions, and it may increase the research funding and uptake of interventions. Journal editors, peer reviewers, and other research sponsors look to clearinghouses for guidance about which research to publish and which future research to support (Burkhardt et al., 2015). Program developers also market their interventions using clearinghouse designations (Neuhoff et al., 2015). Federal, state, and local governments are increasingly mandating the use of evidence-based interventions to inform decision-making (Means et al., 2015). As part of these “tiered-evidence” funding initiatives, clearinghouse ratings have the potential to influence the allocation of billions of dollars appropriated by the federal government (Fagan et al., 2019; Feldman & Haskins, 2016).

## The Need for Clearinghouses to Consider Transparency, Openness, and Reproducibility

Federal clearinghouse standards and processes aim to ensure that interventions reaching their top evidence tiers are truly beneficial by assessing prescribed causal inference methods (e.g., random assignment) to minimize risk of bias (e.g., selection bias). However, studies using these methods often cannot be reproduced, which may be attributable to other limitations related to transparency and openness (Camerer et al., 2016, 2018; Open Science Collaboration, 2015). Notably, growing concerns about missing information in published research give reasons to be cautious about the trustworthiness of intervention research, including “top tier” evidence (McLeroy et al., 2016). These concerns are particularly relevant to evidence clearinghouses because they rely heavily on information published in journal articles and other publicly available manuscripts (Burkhardt et al., 2015; Fagan & Buchanan, 2016; Means et al., 2015; Westbrook et al., 2017; Zack et al., 2019). For example, the Home Visiting Evidence of Effectiveness (HomVEE) review has had to exclude some research due to a lack of information reported in the empirical literature, such as pre-specification of the outcomes of interest (Avellar & Paulsell, 2011). When studies are not registered prospectively, clearinghouses might not know whether all evaluations of a particular intervention have been identified and whether all results of known evaluations are available (Cybulski et al., 2016). If these issues are overlooked, clearinghouses risk disseminating misleading information about intervention effectiveness.

Greater transparency in the design, execution, and analysis of intervention evaluations is paramount to the translation of prevention research into evidence-based policy (Supplee & Meyer, 2015). Although sometimes described as a recent phenomenon, publication and reporting biases have been documented for many decades (Sterling, 1959). Multiple-hypothesis testing and selective non-reporting of results threaten the truthfulness of published research (Goodman et al., 2016). Many studies have shown that journal articles tend to overestimate intervention effects and underestimate their harms (Dwan et al., 2013; Song et al., 2010). Journal articles also often omit information needed to assess external validity or applicability to different populations and settings (Grant et al., 2013; Westbrook et al., 2017). When relevant evidence is missing from clearinghouse reviews because studies are not conducted and reported transparently and openly, even reviews of rigorous studies (e.g., systematic reviews of randomized trials) might reach incorrect conclusions, particularly when those reviews synthesize results by counting the number of positive studies rather than using appropriate methods such as meta-analysis (Valentine et al., 2017).

## Growing Support for Transparency, Openness, and Reproducibility

Concerns about reproducibility have contributed to a long-standing but growing “credibility revolution” across the behavioral, social, and health sciences (Spellman, 2015). As part of this movement, the scientific community is embracing research transparency and openness to the same degree as the study design features promoted by clearinghouse standards of evidence (Baron, 2018). Namely, researchers endorse transparency and openness (Anderson et al., 2007; Christensen et al., 2020), universities have created standard operating procedures (Mayo-Wilson et al., 2018), and journals have implemented new policies (Nosek et al., 2015). Funders also have started requiring open science practices (PCORI Methodology Committee, 2020; Sim et al., 2020; Steinbrook, 2005; Trans-NIH BioMedical Informatics Coordinating Committee, 2020; World Health Organization, 2017) and creating standards related to transparency and reproducibility (Institute of Education Sciences, 2020).

Study registration and comprehensive results reporting provide useful concrete examples of relevant open science practices with widespread support and precedent on which clearinghouse standards of evidence can build. International standards for intervention research reflect the well-established consensus that registration and results reporting are scientifically and ethically imperative. For example, the Declaration of Helsinki states that “[e]very research study involving human subjects must be registered in a publicly accessible database before recruitment of the first subject” and “[r]esearchers have a duty to make publicly available the results of their research on human subjects and are accountable for the completeness and accuracy of their reports” (World Medical World Medical Association, 2013). The World Health Organization developed a widely endorsed minimum dataset for trial registration, and they maintain a list of registries that meet their requirements (De Angelis et al., 2004, 2005).

Because prospective registration has been possible and recommended for decades to reduce selective non-reporting of studies and results (Meinert, 1988; Simes, 1986), multiple federal policies now require this open science practice. The Food and Drug Administration Modernization Act of 1997 (Public Law 105–11) mandated registration of certain trials of medical products, and it authorized the National Institutes of Health (NIH) to create the ClinicalTrials.gov trials registry. Later, the Food and Drug Administration Amendments Act of 2007 (Public Law 110–85) required that many trials report results on ClinicalTrials.gov (Zarin et al., 2016). The Department of Health and Human Services (2016) then issued a final rule implementing registration and results reporting requirements for trials of medical products, and the NIH and Veterans Health Administration (VHA) Office of Research and Development (ORD) adopted complementary policies requiring prospective registration and results reporting for prospective studies evaluating the effects of interventions on health outcomes, including psychological and behavioral interventions (Hudson et al., 2016). The Centers for Medicare and Medicaid Services (2020) requires that trials be registered to receive reimbursement for covered services. Recently, federal departments that run evidence clearinghouses have themselves promoted open science practices, such as prospective study and analysis plan registration, archiving data and materials, and sharing all results publicly in open access reports (Holzwart & Wagner, 2020; Institute of Education Sciences, 2020). To avoid lagging behind and being out of sync with these developments in the scientific ecosystem, clearinghouses should engage directly with this movement toward open science by design. Our study is the first to systematically assess whether and how federal evidence clearinghouses have engaged with a minimum set of open science practices in their standards of evidence.

## Methods

Adapting the Donabedian “structure-process-outcome” model from health services research (Mayo-Wilson et al., 2021), we examined the degree to which the policies, procedures, and practices of federal evidence clearinghouses consider the transparency, openness, and reproducibility of intervention evaluations. This study is part of the Transparency of Research Underpinning Social Intervention Tiers (TRUST) Initiative, a collaboration of intervention scientists that aims to advance open science in social intervention research used to inform evidence-based policy (https://www.trustinitiative.org/).

### Identifying Evidence Clearinghouses

We included clearinghouses that are funded by the US federal government and run by either federal government staff or contracted research organizations (Neuhoff et al., 2015). These clearinghouses’ ratings are highly consequential because they are used to inform policy decisions through evidence-based grantmaking and the awarding of billions of federal prevention dollars. Additionally, federal clearinghouses share similar standards, build upon one another methodologically, and respond to overlapping statutory requirements, political considerations, and research needs. Although they have many similar features, we excluded clearinghouses run by state and local governments (e.g., Washington State Institute for Public Policy), non-governmental organizations (e.g., Blueprints for Health Youth Development), and international clearinghouses (e.g., What Works Centres in the UK).

To be eligible, federal clearinghouses had to include prevention science topics, systematically search the research literature to locate eligible intervention evaluations (Valentine et al., 2017), follow documented standards of evidence for rating interventions (Burkhardt et al., 2015; Means et al., 2015), and post their ratings online (Gough & White, 2018). To identify eligible federal clearinghouses, we reviewed existing literature on evidence clearinghouses (Burkhardt et al., 2015; Davies & Silloway, 2016; Gough & White, 2018; Means et al., 2015; Neuhoff et al., 2015; Paulsell et al., 2017; Valentine et al., 2017; Westbrook et al., 2017), examined federal websites, and consulted with members of the Interagency Federal Evidence Review Workgroup (INFER), a group of federal employees and contractors that met regularly to explore and potentially coordinate shared evidence frameworks and research guidelines across federal agencies.

### Evaluating Federal Clearinghouse Policies, Procedures, and Practices

To evaluate the degree to which federal clearinghouses consider open science, we first obtained information on clearinghouse policies, procedures, and practices. We defined clearinghouse “policies” as the standards of evidence found in clearinghouse manuals and handbooks, “procedures” as the processes and tools used by clearinghouse staff to apply those standards of evidence, and “practices” as the information about interventions that appears online. We obtained information by downloading documents from clearinghouse websites, exploring structured fields of intervention entries on clearinghouse websites, and asking clearinghouse staff to share any relevant information that we did not identify through our review.

Based on the TOP Guidelines (Nosek et al., 2015) and regulations for clinical trials (Drazen et al., 2010; Zarin et al., 2016), we evaluated the degree to which federal clearinghouses consider the following open science practices: citation standards, data sharing, code sharing, materials sharing, design and analysis transparency, study registration, protocol sharing, analysis plan registration, investigator conflicts of interest, public availability of results, and replication. Two authors (SG and EMW) independently copied and pasted verbatim text into a data extraction form, described how the federal clearinghouse consider the relevant open science practice, and resolved discrepancies through discussion.

## Results

We included 10 federal evidence clearinghouses associated with five different divisions of the Departments of Education, Health and Human Services, Justice, and Labor (Table 1). Of these, three clearinghouses are no longer conducting new reviews: Employment Strategies Evidence Review (ESER), Strengthening Families Evidence Review (SFER), and Teen Pregnancy Prevention (TPP) Evidence Review. Because their intervention ratings are still publicly available, we included these clearinghouses in our analysis. Two other clearinghouses became active during our study and were included: Pathways to Work Evidence Review (P2W) and Prevention Services Clearinghouse (PSC). Our evaluation reflects clearinghouse policies, procedures, and practices as of October 2020 (in the cases of active clearinghouses) or the date that the clearinghouse ceased conducting new reviews (in the cases of ESER, SFER, and TPP).

**Table 1:** Federal evidence clearinghouses included in our analysis

Clearinghouse	Federal department	Department division	Relevant legislation and program grants
CLEAR	Labor	Chief Evaluation Office	Reemployment Services and Eligibility Assessment
CrimeSolutions	Justice	Office of Justice Programs	Juvenile Justice Reform Act of 2018
ESER*	Health and Human Services	Administration for Children and Families	Temporary Assistance for Needy Families
HomVEE	Health and Human Services	Administration for Children and Families	Maternal, Infant, & Early Childhood Home Visiting
P2W	Health and Human Services	Administration for Children and Families	Temporary Assistance for Needy Families
PSC	Health and Human Services	Administration for Children and Families	Family First Prevention Services Act
SFER*	Health and Human Services	Administration for Children and Families	Healthy Marriage and Responsible Fatherhood
SPT	Justice	National Gang Center	OJJDP Gang Violence Prevention Programs
TPP*	Health and Human Services	Administration for Children and Families	Teen Pregnancy Prevention Program
WWC	Education	Institute of Education Sciences	Every Student Succeeds Act

*CLEAR* Clearinghouse for Labor and Evaluation Research, *ESER* Employment Strategies Evidence Review, *HomVEE* Home Visiting Evidence of Effectiveness, *OJJDP* Office of Juvenile Justice and Delinquency Prevention, *P2W* Pathways to Work Evidence Clearinghouse, *PSC* Prevention Services Clearinghouse, *SFER* Strengthening Families Evidence Review, *SPT* Strategic Planning Tool, *TPP* Teen Pregnancy Prevention Evidence Review, *WWC* What Works Clearinghouse

^*^This clearinghouse is no longer active

We excluded the Model Programs Guide (MPG) funded by the Department of Justice, Office of Juvenile Justice and Delinquency Prevention, because it utilizes the same standards and database as CrimeSolutions, and was considered redundant. We also excluded the National Registry of Evidence-Based Programs and Practices (NREPP) funded by the Department of Health and Human Services, Substance Abuse, and Mental Health Services Administration because it permanently became publicly inaccessible prior to starting data collection.

### Federal Clearinghouse Policies, Procedures, and Practices Related to Open Science

Overall, we found that seven federal clearinghouses (70%) consider at least one open science practice in at least one of their policies, procedures, or practices (Table 2 and Online Supplement): CrimeSolutions, ESER, Home Visiting Evidence of Effectiveness (HomVEE), P2W, PSC, TPP, and the What Works Clearinghouse (WWC). In order of frequency, the open science practices they consider are replication, public availability of results, investigator conflicts of interest, design and analysis transparency, study registration, and protocol sharing. Three clearinghouses (30%) do not consider any open science practices: Clearinghouse for Labor and Evaluation Research (CLEAR), SFER, and Strategic Planning Tool (SPT). In addition, we found that no clearinghouses consider five open science practices of interest: analysis plan registration, data sharing, code sharing, materials sharing, and citation standards.

**Table 2:** Clearinghouse standards of evidence on the transparency, openness, and reproducibility of intervention evaluations

Practice	Rationale	Clearinghouses	Current standards of evidence
Citation standards	Data, code, and research materials should be cited so that users can audit and validate the results. Crediting authors incentivizes sharing (Bierer et al., 2017).	None	-
Data, code, and materials sharing	Sharing data, code, and research materials guards against incorrect results (e.g., inadvertent mistakes), enables reuse (e.g., to ask new questions about heterogeneity), and helps potential users reproduce interventions in practice (Taichman et al., 2017).	None	-
Design and analysis transparency	To assess risk of bias and applicability, study methods and results should be reported according to CONSORT-SPI (Grant et al., 2018), TREND (Des Jarlais et al., 2004), or other reporting guidelines (Simera et al., 2010): https://www.equator-network.org/.	ESER, HomVEE, WWC	Articulates reporting standards
Study registration	Registering studies in structured, web-based, publicly accessible registries (e.g., www.ClinicalTrials.gov) would help clearinghouses identify all eligible evidence quickly. Prospective registration allows users to evaluate risk of selective non-reporting of studies or results.	HomVEE	Reports registration numbers
		PSC	Prioritizes inclusion of registered studies
Protocol sharing and analysis plan registration	Publicly available protocols and statistical analysis plans (SAPs) allow users to assess whether reported results are consistent with the totality of the evidence from a study (Chan et al., 2013). Because different analysis methods can produce different results, and deviations from planned methods can introduce bias. Reports should describe the nature, timing, and rationale for deviations from planned methods.	PSC	Prioritizes inclusion of studies with published protocols
Investigator conflicts of interest	The presence or absence of conflicts of interest should be declared in all study reports so they are clear to clearinghouses and to other users. (e.g., following the ICMJE Form for Disclosure of Potential Conflicts of Interest)	CrimeSolutions	Prioritizes inclusion of studies without developer involvement
		HomVEE, WWC	Reports developer involvement
Public availability of results	“Secret evidence” undermines public trust. Reports used to rate interventions should be freely available to the public so users can access and appraise the evidence themselves (e.g., open access publication, publication on a preprint server). Public availability would also help clearinghouses identify and obtain relevant evidence research quickly	ESER, HomVEE, P2W, PSC, TPP, WWC	Shares outcome-level data on website in a standardized, tabular format
Replication	To demonstrate that conclusions are reproducible, and therefore more likely to be true, studies showing beneficial effects should be repeated on new analytic samples. Results from multiple studies of the same intervention should be compared using appropriate systematic review and meta-analytic methods that address consistency across studies and risk of selective non-reporting of studies and results (Valentine et al., 2017)	CrimeSolutions, HomVEE, P2W, PSC, WWC	Replication influences ratings
		TPP	Reports whether effects are replicated in multiple studies

*ESER* Employment Strategies Evidence Review, *HomVEE* Home Visiting Evidence of Effectiveness, *P2W* Pathways to Work Evidence Clearinghouse, *PSC* Prevention Services Clearinghouse, *SFER* Strengthening Families Evidence Review, *SPT* Strategic Planning Tool, *TPP* Teen Pregnancy Prevention Evidence Review, *WWC* What Works Clearinghouse

#### Design and Analysis Transparency

Three federal clearinghouses (30%) consider adherence to standards for reporting research design and data analysis. WWC articulates design and analysis transparency standards in their author reporting guides, which provide checklists for reporting evaluations (What Works Clearinghouse, 2018a, b). ESER and HomVEE developed their own reporting guides based on the WWC author reporting guides (Employment Strategies for Low-Income Adults Evidence Review, 2016; Home Visiting Evidence of Effectiveness, 2018a). To some extent, all clearinghouses consider reporting insofar as they assume that design requirements were not met if they were not reported. For example, some clearinghouses require that non-randomized studies demonstrate baseline equivalence between intervention groups, treating studies as if groups were *not* equivalent at baseline when this information is not included in study reports or by contacting study authors. Although related to design and analysis transparency, we did not consider these to be design and analysis transparency requirements because we examined the presence of a codified checklist of reporting standards as described in the TOP Guidelines (Nosek et al., 2015).

#### Study Registration

Two federal clearinghouses (20%) address prospective inclusion of studies in a structured, web-based, publicly accessible registry. While not mentioned in its policies and procedures, HomVEE reports trial registration numbers (when they exist) as part of intervention entries on their website. When there are more than 15 eligible studies for an intervention, PSC assigns points in a manner that prioritizes studies that have been registered (Wilson et al., 2019).

#### Protocol Sharing

One federal clearinghouse (10%) addressed publicly sharing or publishing study protocols. PSC prioritizes studies with protocols as it prioritizes registered studies (Wilson et al., 2019).

#### Investigator Conflicts of Interest

Three federal clearinghouses (30%) address conflicts of interest of intervention evaluators. Both HomVEE and WWC report whether intervention developers were involved in the study (Home Visiting Evidence of Effectiveness, 2018b; What Works Clearinghouse, 2020). In addition, HomVEE reports the funding source for each study (Home Visiting Evidence of Effectiveness, 2018b), while WWC also indicates whether study outcome measures were created by intervention developers (What Works Clearinghouse, 2020). When a program has more than three eligible studies, CrimeSolutions uses independence of the intervention evaluator as one of several criteria to determine the three most rigorous studies to review (CrimeSolutions, 2019b).

#### Public Availability of Results

Six federal clearinghouses (60%) support the public availability of summary results. ESER, HomVEE, P2W, and PSC share outcome-level data using a standardized, tabular format for each intervention entry on their websites. TPP and WWC also provide access to a standardized, tabular database across interventions.

#### Replication

Six federal clearinghouses (60%) consider replication of intervention effects in multiple, non-overlapping samples. TPP examines whether effects in subsequent studies are consistent with an initial study (Mathematica Policy Research, 2016). CrimeSolutions uses a “multiple-studies icon,” and the pattern of findings across multiple studies can influence ratings (CrimeSolutions, 2019a, b). HomVEE, P2W, PSC, and WWC consider whether at least two studies report positive, statistically significant effects (Home Visiting Evidence of Effectiveness, 2018b; Rotz et al., 2020; What Works Clearinghouse, 2020; Wilson et al., 2019).

## Discussion

We found that most federal clearinghouses consider at least one open science practice when reviewing and rating the evidence on intervention effects. However, standards for designating interventions as “evidence-based” incorporate little information about the transparency, openness, and reproducibility of eligible evaluations. Replication is the only practice that is included in standards for determining whether an intervention is “evidence-based,” which is the core mission of evidence clearinghouses, and most clearinghouses do not use meta-analysis or other appropriate methods to estimate intervention effects. Widespread “rule setting” requirements concerning the number of studies with positive results can lead to incorrect conclusions and encourage questionable research practices (Valentine et al., 2017). Moreover, several clearinghouses do not address any open science practices, some clearinghouses consider open science practices only when a certain number of studies are available, and no clearinghouse addresses analysis plan registration, data sharing, code sharing, materials sharing, and citation standards.

Transparency and openness standards have existed for decades, but many studies used by clearinghouses were conducted before it was expected or even possible to adhere to current best practices. By reporting whether existing studies followed best practices, and by requiring that future studies adhere to contemporary standards for transparency and openness to receive their highest ratings, clearinghouses could signal the importance of these standards to the field. Although immediate adoption of all standards at the highest level of implementation seems unlikely, the TOP Guidelines provide a useful framework to help clearinghouses consider which specific standards to adopt and at which level of implementation. Through policies that carefully consider both the lessons learned through prior research, as well as the limitations of legacy studies, and by allowing sufficient lead time for new research to adhere to new transparency requirements, the gradual inclusion of these standards will send a clear message and advance the field of prevention science. Finally, adopting the TOP Guidelines could improve consistency across clearinghouses. It seems likely that clearinghouses might revise their standards at different times for legislative, administrative, and political reasons; nonetheless, federal clearinghouses often build upon each other, and we are optimistic that consistent implementation of some standards could emerge over time.

Although we looked at federal clearinghouses specifically, our recommendations could be implemented by many types of clearinghouses (Buckley et al., 2021). Updating clearinghouse standards could promote more credible and useful intervention research, and thereby improve evidence-informed decision-making at the national, state, and local levels. That is, “evidence-based” interventions would be even more trustworthy if favorable results were found in multiple studies that were registered prospectively, reported comprehensively including materials needed to reproduce their methods, independent of significant conflicts of interest, and computationally reproducible using publicly accessible data and code. Adopting such standards could also encourage investigators who evaluate interventions, and journals that publish intervention research, to adopt open science practices. Although clearinghouses do not have direct control over research conduct, stakeholders design and report studies to meet clearinghouse standards.

Clearinghouses could use the International Committee of Medical Journal Editors (ICMJE) registration policy as a model for supporting future implementation of transparency and openness standards without discarding the existing body of evidence. For example, ICMJE announced in 2004 that studies conducted after 2005 would have to be registered prospectively for publication (De Angelis et al., 2004, 2005). Shortly before ICMJE’s deadline, trial registration increased dramatically (Zarin et al., 2017). Similarly, clearinghouses could incentivize transparency, and they could identify limitations in current evidence, by requiring that evaluations initiated after a future date be registered prospectively to be eligible for clearinghouse review. Greater transparency and openness would also benefit clearinghouses directly by making it easier to obtain the information needed to identify what works, for whom, and under what conditions.

### Taking up TOP at Evidence Clearinghouses

To facilitate clearinghouse promotion of research transparency and reproducibility, we used the modular format of the TOP Guidelines (Nosek et al., 2015) to identify open science practices that clearinghouse can implement (see Table 3). Clearinghouses could coordinate implementation to promote consistency across scientific disciplines and reports, or clearinghouses might adopt different standards at different levels over time to meet their specific needs. At a minimum, clearinghouses could collect data on and report whether intervention evaluations used open science practices. This information could be visualized using badges that acknowledge and signal the use of open practices in intervention evaluations (Kidwell et al., 2016). At a higher level of implementation, clearinghouses could include open science practices in their standards of evidence. For example, prospective registration could influence whether an intervention is eligible to receive a “top tier” rating. As with standards for study design features, clearinghouses might assume that open science practices were not used if they were not reported. At the highest level of implementation, clearinghouses could require and verify that evaluations used open science practices. Verifying standards might require different levels of effort. It might be time consuming to compare reported outcomes with analysis plan registrations to confirm whether results for all planned outcomes and analyses have been reported. It might be even more resource-intensive to use publicly available data and code to reproduce reported results. Coordinated efforts with partner journals and funders—such as a Registered (Replication) Reports model—could help with verifying these practices and thereby reduce the direct burden on clearinghouses (Chambers, 2019). Higher levels of implementation might be more feasible as funders require and provide sufficient resources to investigators to include open science practices in their studies, such as NIH requirements that grantees plan prospectively for managing and sharing data from all NIH-funded projects (Collins, 2020).

**Table 3:** Proposed implementation of Transparency and Openness Promotion Guidelines by evidence clearinghouses

Standard	Levels of implementation (from least to most effort required for implementation)
Citations standards	1.* Report* whether studies cited the data, code, and research materials used to produce their results 2*. Rating is influenced by* data, code, and research material citations (e.g., appropriate citations improve intervention rating) 3*. Verify* adequate data, code, and material citations (e.g., confirm that DOIs and URLs lead to these items to obtain “top tier” rating)
Data, code, and materials sharing	1.* Report* whether data, code, and research materials are publicly available (e.g., provide links to data, code, and materials if available) 2.* Rating is influenced by* data, code, and materials sharing (e.g., sharing in trusted repositories improves intervention rating) 3.* Verify* findings by reproducing the results using data, code, and materials (e.g., verification required to obtain “top tier” rating)
Design and analysis transparency	1.* Report* whether study reports adhered to reporting guidelines (e.g., articles includes completed CONSORT-SPI checklist) 2.* Rating is influenced by* adherence to reporting guidelines (e.g., recommended information improves intervention rating) 3.* Verify* study reports contain minimum recommended information (e.g., confirm information is present to obtain “top tier” rating, or publicly share any missing information obtained through contacting authors)
Study registration	1.* Report* whether studies were registered, and whether they were registered completely and prospectively 2.* Rating is influenced by* study registration status (e.g., prospective registration improves intervention rating) 3.* Verify* complete, prospective study registration (e.g., confirm all registered outcomes are reported as registered, or deviations are adequately described, to obtain “top tier” rating)
Protocol sharing and analysis plan registration	1.* Report* whether studies publicly shared a protocol and registered their analysis plan 2.* Rating is influenced by* protocol and analysis plan sharing (e.g., publishing a protocol or analysis plan improves intervention rating) 3.* Verify* quality of protocol sharing and analysis plan (e.g., confirm protocols and analysis plans match study reports, or deviations are adequately described to obtain “top tier” rating)
Investigator conflicts of interest	1.* Report* whether study investigators had conflicts of interest 2.* Rating is influenced by* investigator conflicts of interest (e.g., declarations of interest improve intervention rating) 3.* Verify* all conflicts of interest are declared (e.g., confirm information required by ICMJE Disclosure Forms is declared to obtain “top tier” rating, or share conflicts of interest obtained through contacting authors)
Public availability of results	1.* Report* whether study results are publicly available in a database using a standardized, tabular format 2.* Rating is influenced by* public availability of results (e.g., entry in ClinicalTrials.gov results database improves intervention rating) 3.* Verify* all study results are publicly available (e.g., confirm whether all results included in ratings are in the ClinicalTrials.gov results database to obtain “top tier” rating, or publicly share unpublished information obtained through contacting authors)
Replication	1.* Report* whether favorable intervention effects were replicated with non-overlapping samples 2.* Rating is influenced by* replication of intervention effects (e.g., an independent replication improves intervention rating) 3.* Verify* replication of effects (e.g., confirm consistency through systematic reviews and meta-analyses to obtain “top tier” rating)

*CONSORT-SPI* Consolidated Standards of Reporting Trials for Social and Psychological Interventions, *DOI* digital object identifier, *ICMJE* International Committee of Medical Journal Editors, *URL* Uniform Resource Locator

Clearinghouses should also consider ways in which their current standards encourage questionable research practices such as multiple-hypothesis testing and selective non-reporting of studies and results. For example, clearinghouses that select only certain studies from the evidence base, or that allow users to search for “positive” results, present evidence in a way that might systematically overestimate the effectiveness of those interventions and undermine the goals of replication. To mitigate these problems, evidence-based policy should be based on systematic reviews and meta-analyses (Valentine et al., 2017). These methods are appropriate for evidence clearinghouse because they can identify replications, group multiple reports about the same study to ensure that studies and participants are not “double-counted” (Mayo-Wilson et al., 2017), and synthesize results using formal, statistically appropriate methods. Clearinghouses would have to consider which studies and outcomes to include in meta-analyses, and how to account for study limitations in their inclusion criteria and interpretation of findings. For example, systematic reviews vary in whether they include results from all studies in meta-analyses or include only those studies meeting specific standards (e.g., regarding risk of bias). Clearinghouses should explore developing standards for study eligibility, handling missing data, assessing risk of bias, and sensitivity analyses following best-practices for research synthesis (Higgins et al., 2019). Confidence in evidence could then be rated using internationally accepted standards that consider consistency across replications, such as GRADE (Montgomery et al., 2019).

### Strengths and Limitations

Strengths of this study include the use of formal open science frameworks and two independent assessors for data extraction. It is a limitation that the TOP Guidelines were designed for journal policies and had to be adapted for clearinghouses, and further feedback from the community might identify ways to refine our approach for this purpose. Although they have many things in common, federal clearinghouses do sometimes respond to different legislative mandates and political considerations, which can contribute to differences between clearinghouses in their ratings of similar evidence. Adopting transparency standards that focus on research processes and practices, rather than impact designs or specific statutory outcomes, could provide an opportunity to improve consistency across clearinghouses. It is also a limitation that we excluded non-federal clearinghouses. We focused on federal clearinghouses because their intervention ratings are used to inform social policy decisions through evidence-based grantmaking and tiered-evidence legislation. Even if we had been able to define and to identify all local, national, and international clearinghouses, we did not have resources to evaluate them. Given the influence of federal clearinghouses on other clearinghouse standards of evidence, and given the emphasis across clearinghouses on study design features, we expect our findings would be similar if we had included a larger sample of non-federal clearinghouses. That is, we expect that we would have found little consideration for the transparency and reproducibility of intervention evaluations in clearinghouse standards of evidence. Moreover, our recommendations for implementing the TOP Guidelines could be used by federal and non-federal clearinghouses alike.

## Conclusion

To our knowledge, this is the first study to examine the degree to which clearinghouse policies, procedures, and practices are aligned with standards for transparency, openness, and reproducibility. Although clearinghouses consider the rigorousness of study designs, we found that clearinghouse standards of evidence do not reflect current best practices for transparency and openness in study design, conduct, and reporting. Consequently, “top tier” evidence might be misleading because some “evidence based” interventions might be based on false positive results. Clearinghouses could reduce the likelihood of drawing incorrect conclusions for policy and practice by incorporating transparency and openness in their standards of evidence. Having identified opportunities for improvement, we provide concrete recommendations to update clearinghouse standards for designating interventions as “evidence-based” (Table 3). There is international consensus that open science practices should be considered when evaluating intervention effects, judging confidence in a body of evidence, and making recommendations. These practices have been integrated into international research guidelines, federal laws and regulations, and policies of journals, funders, universities, and academic societies. We encourage clearinghouses to synchronize with other stakeholders in this movement toward a more open science that facilitates transparent intervention research for evidence-based policy and practice.

## Supplementary Information

Below is the link to the electronic supplementary material.

Supplementary file1 (DOCX 42 KB)
