# Supplementary material for: Clearinghouse Standards of Evidence on the Transparency, Openness, and Reproducibility of Intervention Evaluations
Source: Prev Sci. 2021 Aug 6;23(5):774–86. doi: 10.1007/s11121-021-01284-x (PMC9283145; doi:10.1007/s11121-021-01284-x)
Supplement: Supplementary file 1 — Supplementary file1 (DOCX 42 KB) [file 11121_2021_1284_MOESM1_ESM.docx]

**Clearinghouse standards of evidence on the transparency, openness, and reproducibility of intervention evaluations**

| **Open Science Practice** | **Clearinghouse** | **Standard of Evidence** | **Clearinghouse Policy, Procedure, or Practice** |
| --- | --- | --- | --- |
| Citation Standards | None | - | - |
| Data Sharing | None | - | - |
| Code Sharing | None | - | - |
| Materials Sharing | None | - | - |
| Analysis Plan Registration | None | - | - |
| Protocol Sharing | PSC | Prioritizes inclusion of studies with published protocols | “For programs and services with more than 15 eligible studies, a point system will be used to determine the order of studies reviewed. When a study is determined to be eligible for review using the above-described criteria, reviewers assign points to studies as follows.... Pre-Registered Study Designs. 3 points for studies that ... have published study protocols. Points are totaled for each study (maximum of 12 points). Studies are then sorted by the summed point total and reviewed in that order.”^1^ |
| Study  Registration | HomVEE | Reports study registration numbers | Example from the website “Study Registration: Clinicaltrials.gov Identifier: NCT02093052. Study registration was assessed by HomVEE beginning with the 2014 review.”^2^ |
|  | PSC | Prioritizes inclusion of registered studies | “For programs and services with more than 15 eligible studies, a point system will be used to determine the order of studies reviewed. When a study is determined to be eligible for review using the above-described criteria, reviewers assign points to studies as follows.... Pre-Registered Study Designs. 3 points for studies that were pre-registered in a trial registry…. Points are totaled for each study (maximum of 12 points). Studies are then sorted by the summed point total and reviewed in that order.”^1^ |
| Investigator  Conflicts of Interest | CrimeSolutions | Prioritizes inclusion of studies without developer involvement | “The criteria used to determine the three most rigorous studies include … independence of evaluator.”^3^ |
|  | HomVEE | Reports whether program developers were involved in the study | “Evaluator Independence. HomVEE reported the funding source for each study and whether any of the study authors were model developers.”^4^ |
|  | WWC | Reports whether program developers were involved in the study | “The WWC does not exclude studies conducted or outcomes created by the developer of the product being reviewed. The authors of all studies are indicated in WWC reports, and the WWC indicates the source of all outcome measures that are used, including those created by the developer.”^5^ |
| Design and Analysis Transparency | ESER | Articulates reporting standards | “This document provides recommendations for how to describe randomized controlled trials and matched comparison group design studies and report their findings in a way that is clear, complete and transparent…. The ESER team produced this reporting guide to recommend best practices for describing impact evaluations of employment programs and strategies. Authors of future evaluation reports may find this reporting guide to be a useful reference tool.”^6^ |
|  | HomVEE | Articulates reporting standards | “This document provides guidance about how to describe randomized controlled trials and matched comparison group design studies and report their findings in a way that is clear, complete and transparent. Reporting the information described below is considered a best practice in general, but can also help reviewers from the Home Visiting Evidence of Effectiveness (HomVEE) review assess the appropriate rating to assign to the study.”^7^ |
|  | WWC | Articulates reporting standards | “This document provides guidance to study authors about how to describe regression discontinuity design studies and report their findings in a way that is clear, complete, and transparent.”^8^ |
|  |  |  | “This document provides guidance to study authors about how to describe group design studies and report their findings in a way that is clear, complete, and transparent.”^9^ |
| Public Availability  of Results | ESER | Shared outcome-level data on website in a standardized, tabular format | “Reviewers systematically recorded information about a study, and about selected outcomes within the study, in a template. The templates were combined into a database of results that is searchable on the review’s website.”^10^ |
|  | HomVEE | Shares outcome-level data on website in a standardized, tabular format | Effects are shown in a standardized, tabular format in the “Research & Outcome Measure Details: Show Findings Details” section of intervention entries.^11^ |
|  | P2W | Shares outcome-level data on website in a standardized, tabular format | “Each intervention also has a dedicated web page, clearly indicating the findings … from each of the outcome domains.”^12^ |
|  | PSC | Shares outcome-level data on website in a standardized, tabular format | “The individual findings from each contrast with a high or moderate rating are reported on the Prevention Services Clearinghouse website.”^13^ |
|  | TPP | Shared outcome-level data in a file with a standardized, tabular format | See “*Effect Size Table for Online Final*.*xlsx*.”^14^ |
|  | WWC | Shares outcome-level data on website in a standardized, tabular format | “The WWC provides the capability for users to extract data resulting from WWC reviews for every study that meets standards… as part of our open data approach to increase transparency.”^15^ |
| Replication | CrimeSolutions | Replication influences program ratings | “A multiple-studies icon … depicts programs that have more than one study in the evidence base that demonstrates effects in a consistent direction…. There is greater evidence supporting the rating because with each replication it becomes less likely results were due to something other than the program.”^3^ |
|  |  |  | “In some cases, multiple meta-analyses provide information about the same outcome. For each meta-analysis, that outcome is first rated separately for quality and validity … and then classified according to the five classes.”^16^ |
|  | HomVEE | Replication influences program ratings | “HHS criteria for evidence-based early childhood home visiting service delivery: models must meet at least one of the following criteria... at least two high- or moderate-quality impact studies of the model using non-overlapping analytic study samples find one or more favorable, statistically significant impacts in the same domain.”^4^ |
|  |  |  | “HomVEE classified impacts as replicated if favorable, statistically significant impacts were shown in the same outcome domain in at least two non-overlapping analytic study samples.”^4^ |
|  | P2W | Replication influences program ratings | “Interventions receiving the Well-Supported rating…. Findings rated high or moderate from at least two studies conducted in the United States must show favorable and statistically significant effects, with no strong countervailing evidence, for this rating to be assigned.”^12^ |
|  | PSC | Replication influences program ratings | “A program or service is rated as a well-supported practice if it has at least two contrasts with non-overlapping samples in studies carried out in usual care or practice settings … that achieve a rating of moderate/high on design and execution and demonstrate favorable effects in a target outcome.”^1^ |
|  | TPP | Reported whether program effects are replicated in more than one study | “When multiple studies examine program impacts on a common outcome, the review team records the effect size and associated confidence interval reported in the first study establishing the program’s evidence of effectiveness. For subsequent studies, the review team then examines whether the effect sizes reported in these studies fall within the confidence interval reported in the initial study.”^17^ |
|  | WWC | Replication influences program ratings | “WWC characterization of findings in intervention reports. Positive effects: At least two studies are rated Meets WWC Standards Without Reservations or Meets WWC Standards With Reservations.”^5^ |
|  |  |  | “Criteria used to determine extent of evidence for an intervention. Medium to large: The domain includes more than one study.”^5^ |
|  |  |  | “Levels of evidence for practice guide recommendations. Requirement Strong evidence base: The research includes studies that meet WWC standards and provide a “medium to large” extent of evidence.”^5^ |

Abbreviations: ESER: Employment Strategies Evidence Review. HomVEE: Home Visiting Evidence of Effectiveness. P2W: Pathways to Work Evidence Clearinghouse PSC: Prevention Services Clearinghouse. SFER: Strengthening Families Evidence Review. SPT: Strategic Planning Tool. TPP: Teen Pregnancy Prevention Evidence Review. WWC: What Works Clearinghouse

References

1. Wilson SJ, Price CS, Kerns SEU, Dastrup SD, Brown SR. *Title IV-E Prevention Services Clearinghouse Handbook of Standards and Procedures, version 1.0, OPRE Report # 2019-56.* Washington, DC: Office of Planning, Research, and Evaluation, Administration for Children and Families, U.S. Department of Health and Human Services; 2019.

2. Home Visiting Evidence of Effectiveness. Entry for “Bernard, K. (2014). Neurobiology of maternal sensitivity and delight among high-risk mothers: An event-related potential study. ProQuest Dissertations & Theses, 1459753664.”. <https://homvee.acf.hhs.gov/study-detail?title=WWHV049895>.

3. CrimeSolutions. How We Review and Rate a Program From Start to Finish. 2019; <https://www.crimesolutions.gov/about_starttofinish.aspx>. Accessed 12 December, 2018.

4. Home Visiting Evidence of Effectiveness. *Review Process.* Washington, DC: Office of Planning, Research and Evaluation, Administration for Children and Families, U.S. Department of Health and Human Services; 2018.

5. What Works Clearinghouse. *Procedures Hanbook (Verion 4.1).* Washington, DC: Institute of Educational Sciences, U.S. Department of Education; 2020.

6. Employment Strategies for Low-Income Adults Evidence Review. *Reviewing the Research on Improving Employment Outcomes for Low-Income Adults.* Washington, DC: Office of Planning, Research and Evaluation, Administration for Children and Families, U.S. Department of Health and Human Services; 2015.

7. Home Visiting Evidence of Effectiveness. *Home Visiting Evidence of Effectiveness Reporting Guide for Study Authors* Washington, DC: Office of Planning, Research and Evaluation, Administration for Children and Families, U.S. Department of Health and Human Services; 2018.

8. What Works Clearinghouse. *Reporting Guide for Study Authors: Regression Discontinuity Design Studies (Version 1.0).* Washington, DC: Institute of Educational Sciences, U.S. Department of Education; 2018.

9. What Works Clearinghouse. *Reporting Guide for Study Authors: Group Design Studies (Version 2.0).* Washington, DC: Institute of Educational Sciences, U.S. Department of Education; 2018.

10. Mastri A, Sama-Miller E, Clarkwest A. *Employment Strategies for Low-Income Adults Evidence Review: Standards and methods. OPRE Report 2015-27.* Washington, DC: Office of Planning, Research and Evaluation, Administration for Children and Families, U.S. Department of Health and Human Services; 2015.

11. Home Visiting Evidence of Effectiveness. Entry for “Lowell, D. I., Carter, A. S., Godoy, L., Paulicin, B., & Briggs‐Gowan, M. J. (2011). A randomized controlled trial of Child FIRST: A comprehensive home‐based intervention translating research into early childhood practice. Child development, 82(1), 193-208.". <https://homvee.acf.hhs.gov/study-detail?nid=49583>.

12. Rotz D, Sama-Miller E, Burkander P. *Protocol for the Pathways to Work Evidence Clearinghouse: Methods and Standards. OPRE Report # 2020-44.* Washington, DC: Office of Planning, Research, and Evaluation, Administration for Children and Families, U.S. Department of Health and Human Services; 2020.

13. Prevention Services Clearinghouse. Entry for “Brief Strategic Family Therapy”. <https://preventionservices.abtsites.com/programs/201/show>.

14. Teen Pregnancy Prevention Evidence Review. Effect Size Table for Online Final.xlsx. <https://tppevidencereview.youth.gov/Excel/Effect%20Size%20Table%20for%20Online%20Final.xlsx>.

15. What Works Clearinghouse. Data from individual studies. <https://ies.ed.gov/ncee/wwc/StudyFindings>.

16. CrimeSolutions. How We Review and Rate a Practice From Start to Finish. 2019; <https://www.crimesolutions.gov/about_practicereview.aspx>. Accessed 12 December, 2018.

17. Mathematica Policy Research. *Identifying Programs that Impact Teen Pregnancy, Sexually Transmitted Infections, and Associated Sexual Risk Behaviors. Review Protocol Version 5.0.* Washington, DC: Office of the Assistant Secretary for Planning and Evaluation, U.S. Department of Health and Human Services; 2016.
